# Numerically Efficient Three-Dimensional Model for Non-Linear Finite Element Analysis of Reinforced Concrete Structures

**DOI:** 10.3390/ma14071578

**Published:** 2021-03-24

**Authors:** Sławomir Dudziak

**Affiliations:** Building Structures, Geotechnics and Concrete Department, Building Research Institute (ITB), ul. Filtrowa 1, 00-611 Warszawa, Poland; s.dudziak@itb.pl

**Keywords:** non-linear finite element analysis, reinforced concrete structures, tension stiffening effect, engineering application

## Abstract

The paper concerns the non-linear finite element analysis (NLFEA) of Reinforced Concrete (RC) structures for engineering applications. The required level of complexity of constitutive models for such analysis was discussed and non-linear elastic models combined with the smeared cracking approach proved to be efficient. A new constitutive hypoelastic-brittle model of concrete based on these assumptions was proposed. Moreover, a method including the tension stiffening effect (TS) was developed. This phenomenon is connected with the bond properties between concrete and steel and, in some situations, has significant influence on the deflections of RC structures. It is often neglected by or included in the constitutive model of concrete. In the paper, an alternative approach was presented, in which this phenomenon is taken into account by generalising the material model of reinforcing steel. This approach is consistent with modern design standards and has solid physical foundations. The proposed models were implemented in the Abaqus code via UMAT user’s procedure coded in FORTRAN. Model verification and validation were presented in four case studies, concerning: a Willam’s test (examination on material point level), a beam with bending failure, and two beams with shear failure (with and without stirrups). The obtained results were compared with experimental outcomes and numerical results obtained by other researchers. The presented approach enables the accurate prediction not only of load capacity but of structural deformability, due to the precise description of TS. Thus, it promises to be a useful engineering tool.

## 1. Introduction

Non-linear finite element (NLFEA) analysis of reinforced concrete (RC) structures plays an increasingly important role in engineering applications [1,2]. It can be used in the design process of complex structures [3,4], in the evaluation of different scenarios of retrofitting deteriorated structures [5,6,7] (in such analyses the appropriate model for interface between strengthening elements and existing structures is also important), in the assessment of the safety of structures, which were designed according to outdated standards [8], in the proccess of desiging structural health monitoring systems [9] or to assess innovative materials and structures performance [10]. The proposal of reasonable constitutive models of behaviour of reinforced concrete is crucial to making non-linear analysis an everyday tool for structural engineers. Such models should satisfy two main conditions. On the one hand, they must reproduce correctly concrete behaviour in triaxial stress state in possibly wide loading conditions. On the other hand, they should be numerically efficient to enable the use of personal computers during NLFEA. Additionally, it is recommended to use models in which their parameters have clear physical meaning and can be calibrated with the results of standard material strength tests [11].

Many constitutive models of RC structures have been proposed, but only few fulfil all of the above conditions. In scientific research supported by numerical analyses, the constitutive models formulated in the framework of the theory of plasticity are the most common [12]. In order to describe the behaviour of concrete in triaxial stress state, the non-associated flow rule is necessary [13,14], which results in an unsymmetrical tangent stiffness matrix. Consequently, the analysis of large-scale structures using these models is time-consuming, so their applicability in engineering applications is quite limited. Moreover, they demand the calibration of the shape of the plastic potential, which is not an easy task, using the results of standard material strength tests [15].

Recent papers show that models based on non-linear elasticity and the smeared crack concept can be useful in the analysis of large building structures. Spiliopoulos and Lykidis [16] proposed an efficient non-linear model of RC structures for dynamic analysis, based on Kotsovos’ constitutive law [17]. They demonstrate its efficiency with a dynamic analysis of a shear wall and an RC frame. Markou and Papadrakakis [18] created the ReConAn software, which is based on the modified Kotsovos law. They showed its engineering applicability by the analysis of, e.g., an RC bridge [19]. Mourlas et al. have adapted the Kotsovos model to non-monotonic loads by the introduction of: an efficient cracks closure algorithm [20] and damage factors [21]. Engen et al. [22] implemented this model in the DIANA code and showed its usefulness by the simulation of the behaviour of a large offshore shell structure. Lewinski and Wiech [23] proposed a modified Kotsovos model for the analysis of moderate-thickness RC plates.

The common feature of most of the mentioned models is that they neglect the tension stiffening effect (TS) due to modelling the behaviour of concrete as fully brittle. TS is a phenomenon of tensile stresses in concrete in cross-sections between cracks, due to the bond of rebar to concrete, increasing the overall stiffness of the RC element [24]. Usually, it has limited significance for load capacity, but can be important during the assessment of serviceability limit states. Its influence on the stiffness of structural elements depends mostly on: reinforcement ratio and bond quality (rebar type, duration of load) [25]. In elasto-plastic models, it is most often included by a descending branch after crack formation. In this approach, the shape of the descending branch is rarely related to the reinforcement ratio or the quality of the bond [26]; thus, this way of modelling the phenomenon lacks physical justification.

**Figure 1:**
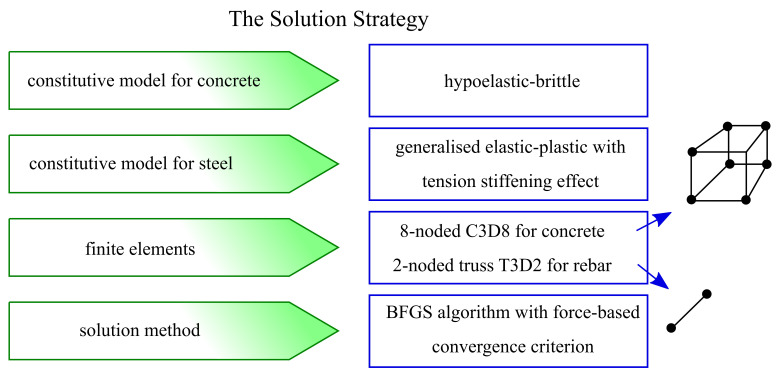
Summary of proposed solution strategy.

In the present paper, a new solution strategy for NLFEA of large-scale RC structures is proposed. It is based on: variable tangent moduli of the constitutive law for concrete in the compression range and the non-orthogonal smeared crack concept in the tensile regime and multi-linear generalised model of reinforcement steel, which cover the TS effect in a way consistent with design standards and the theory of RC structures. These models were implemented in the Abaqus code [27] via UMAT user’s procedure. Main features of the proposed solution strategy were summarised in Figure 1. Model verification and validation are shown in examples. Its accuracy and efficiency is compared with other models, which are described in the literature.

## 2. Proposed Solution Strategy

### 2.1. Constitutive Model of Concrete

In order to describe the behaviour of concrete, it is convenient to decompose stress and strain state into deviatoric and hydrostatic components and express them by octahedral coordinates [17]. Therefore, octahedral stresses ($\sigma_{oct}$, $\tau_{oct}$) and strains ($\epsilon_{oct},\gamma_{oct}$) are used to formulate the proposed deformational model:

(1) $$\begin{array}{c} \sigma_{oct}=\frac{1}{3}\left(\sigma_{1}+\sigma_{2}+\sigma_{3}\right)\\ \tau_{oct}=\frac{1}{3}\sqrt{{\left(\sigma_{1}-\sigma_{2}\right)}^{2}+{\left(\sigma_{2}-\sigma_{3}\right)}^{2}+{\left(\sigma_{3}-\sigma_{1}\right)}^{2}}\\ \epsilon_{oct}=\frac{1}{3}\left(\epsilon_{1}+\epsilon_{2}+\epsilon_{3}\right)\\ \gamma_{oct}=\frac{2}{3}\sqrt{{\left(\epsilon_{1}-\epsilon_{2}\right)}^{2}+{\left(\epsilon_{2}-\epsilon_{3}\right)}^{2}+{\left(\epsilon_{3}-\epsilon_{1}\right)}^{2}}, \end{array}$$

where: $\sigma_{i}$, $\epsilon_{i}$—principal stresses and strains (i=1, 2, 3). In the present paper, hypoelastic relations are proposed to characterise concrete deformability. Incremental hydrostatic and deviatoric responses are described with the following relations:

(2) $$\begin{array}{c} d\sigma_{oct}=3K_{t}\left(\gamma_{oct}\right)\;d\epsilon_{oct}\\ d\tau_{oct}=G_{t}\left(\gamma_{oct}\right)\;d\gamma_{oct}, \end{array}$$

where: $K_{t}$—the tangent bulk modulus, and $G_{t}$—the tangent shear modulus. It is assumed that shear strain has the most significant influence on tangent modules. Consequently, the $K_{t}$ and $G_{t}$ modules change is described by the same function:

(3) $$\frac{G_{t}}{G_{0}}=\frac{K_{t}}{K_{0}}=\frac{1-A\gamma_{oct}+C\gamma_{oct}^{3}}{{\left(1-A\gamma_{oct}+B\gamma_{oct}^{2}-C\gamma_{oct}^{3}\right)}^{2}}.$$

Parameters *A*, *B*, and *C* were calibrated for normal strength concrete with the test results [28]:

(4) $$A=187-0.752f_{c}\;B=\left(2.32-0.0549f_{c}\right)\cdot 10^{6},$$

(5) $$C=\left(172-4.29f_{c}\right)\cdot 10^{6},$$

where $f_{c}$—the concrete compressive strength substituted in MPa. The same function was used in Reference [29] to associate the tangential Young modulus with equivalent uniaxial strain. The model is formulated for stress and strains increments, so it can be considered as hypoelastic. A detailed discussion on such constitutive models can be found in Reference [12].

**Figure 2:**
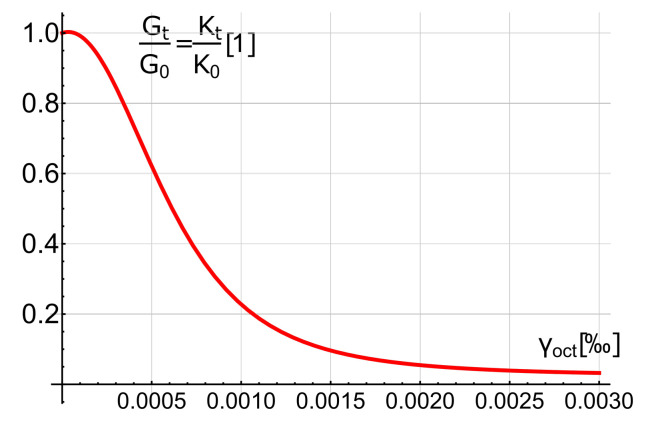
Variation of tangent moduli with octahedral shear strain.

**Figure 3:**
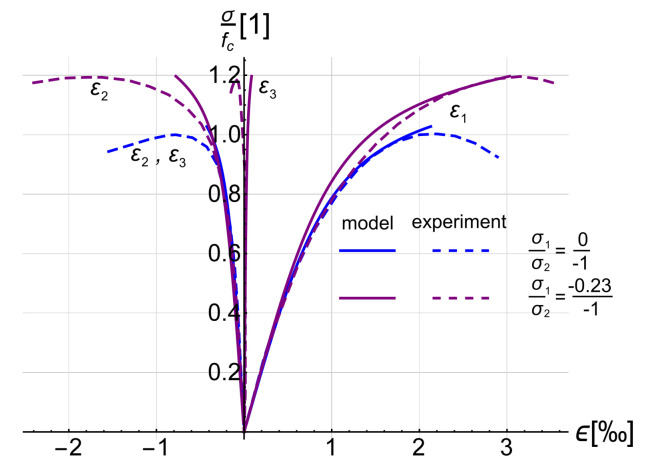
Comparison of theoretical and experimental curves σ − ϵ.

It is well known that hydrostatic stress has little influence on the shear modulus, whereas the shear state induces volume changes, as well (this is called deviatoric-hydrostatic coupling) [17,28]. Moreover, the ultimate state dilatation phenomenon occurs, which causes increase in the volume of the material despite the compressive stress state [15,17], and the Poisson ratio grows up to 0.5. The proposed model does not cover these effects and predicts a constant Poisson ratio. The main non-linear effect determining the behaviour of RC structures, however, is cracking, and the above-mentioned effects play an important role just before reaching load capacity. The examples presented in the following section of the paper will show that the proposed model is sufficient to capture the behaviour of RC structures on a global level. It is worth emphasising that structures designed according to the standards [30,31] should fail due to the yielding of reinforcing steel. Consequently, compression states with high values of hydrostatic stress, in which much more complex material models are needed, are quite rare in real structures. It is worth mentioning that even simpler material models are sometimes applied to some complex, in terms of computational effort, problems [32]. The function $G_{t}$ – $\gamma_{oct}$ is presented in Figure 2, whereas the comparison of analytical and experimental test results [33] is presented in Figure 3.

### 2.2. Ultimate Surface of Concrete

In general, the ultimate surface defines stress states that are admissible, and allows to assess material effort [34]. For the admissible stress states inside the ultimate surface, the function assumes negative values, and, for states outside the surface, it assumes positive values. The results of experimental studies show that the surface of the concrete depends on three invariants of the stress tensor [35]. In the present paper, the *PJ* criterion proposed by Podgorski was used [36,37]. This criterion is formulated in octahedral ($\sigma_{oct}$, $\tau_{oct}$) and Haigh-Westergaard coordinates (*r*, φ, *h*). The relations for the former coordinates can be found in the previous section, whereas formulas for the latter are given below:

(6) $$\begin{array}{c} r=\sqrt{2J_{2}}=\sqrt{3}\;\tau_{oct}\\ \cos\left(3\varphi \right)=J\left(\varphi \right)=\frac{3\sqrt{3}\;J_{3}}{2{\left(J_{2}\right)}^{\frac{3}{2}}}\\ h=\frac{I_{1}}{\sqrt{3}}=\sigma_{oct}\sqrt{3}\;, \end{array}$$

where: $I_{1}=\sigma_{1}+\sigma_{2}+\sigma_{3}$—the first stress invariant, $J_{2}=\frac{1}{3}\left(\sigma_{1}^{2}+\sigma_{2}^{2}+\sigma_{3}^{2}-\sigma_{1}\sigma_{2}-\sigma_{2}\sigma_{3}-\sigma_{1}\sigma_{3}\right)$—the second deviatoric stress invariant, and $J_{3}=\left(\sigma_{1}-\sigma_{oct}\right)\left(\sigma_{2}-\sigma_{oct}\right)\left(\sigma_{3}-\sigma_{oct}\right)$—the third deviatoric stress invariant.

The deviatoric section is described by the following formulas:

(7) $$r=\frac{1}{P(J(\varphi ))}\;P\left(J\left(\varphi \right)\right)=\cos\left[\frac{1}{3}\arccos\left(\alpha J\left(\varphi \right)\right)-\beta \right]\;,$$

where 0≤α≤1 and $0\leq \beta \leq \frac{\pi }{6}$ are parameters for calibration.

**Figure 4:**
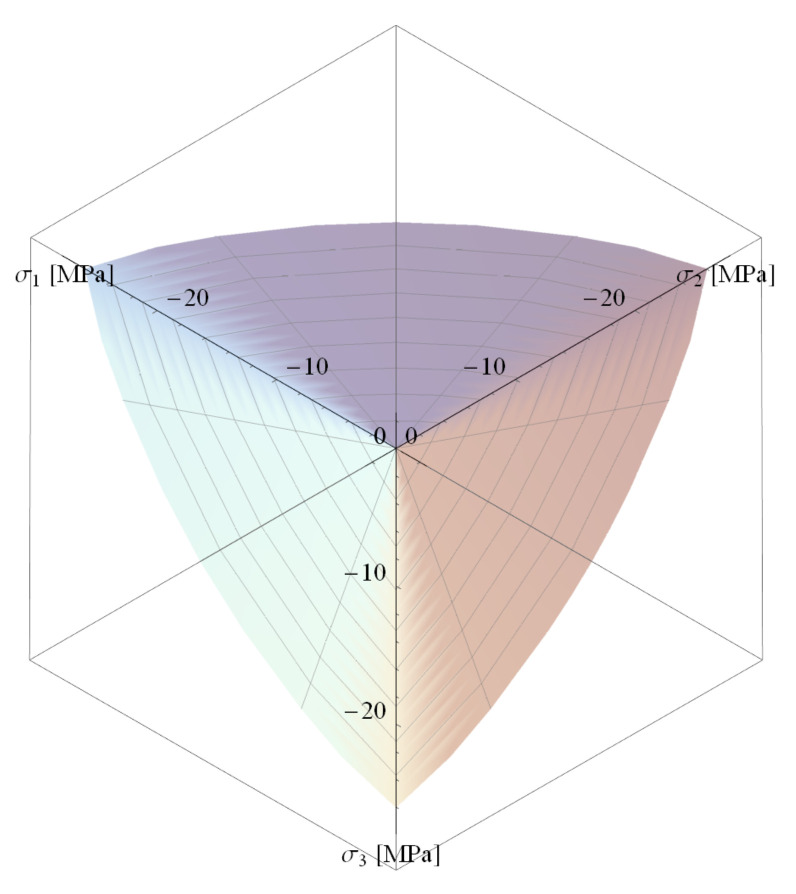
The deviatoric section of the PJ surface.

For positive and low values of hydrostatic stress, the deviatoric section has a shape close to triangular, and, as the value of this stress decreases, the shape becomes more curved (see Figure 4). In order to describe the shape of the deviatoric section correctly, the radius r(φ) ratios for different φ are introduced:

(8) $$\lambda =\frac{r(0)}{r(\frac{\pi }{3})}\;\theta =\frac{r(\frac{\pi }{6})}{r(\frac{\pi }{3})}\;.$$

According to Podgorski, the coefficients α and β can be obtained based on λ and θ in an iterative manner. Formulas for the accurate and direct calculation of these parameters were derived by Lewinski [38], and their usefulness and correctness was confirmed by the author of the surface [39]. They have the following form:

(9) $$\alpha =\sin\left(3\alpha_{0}\right)\;\beta =\frac{\pi }{6}-\arctan\left[\frac{\theta \left(\frac{1}{\lambda }-1\right)}{2\sin\alpha_{0}}\right]$$

(10) $$\alpha_{0}=\arccos\left[\frac{\theta }{2}\left(1+\frac{1}{\lambda }\right)\right]\;.$$

The surface meridian in octahedral coordinates is described by a second-degree polynomial:

(11) $$F\left(\sigma_{oct},\tau_{oct},\varphi \right)=\sigma_{oct}-C_{0}+C_{1}P\left(J\right)\tau_{oct}+C_{2}\tau_{oct}^{2}=0.$$

**Figure 5:**
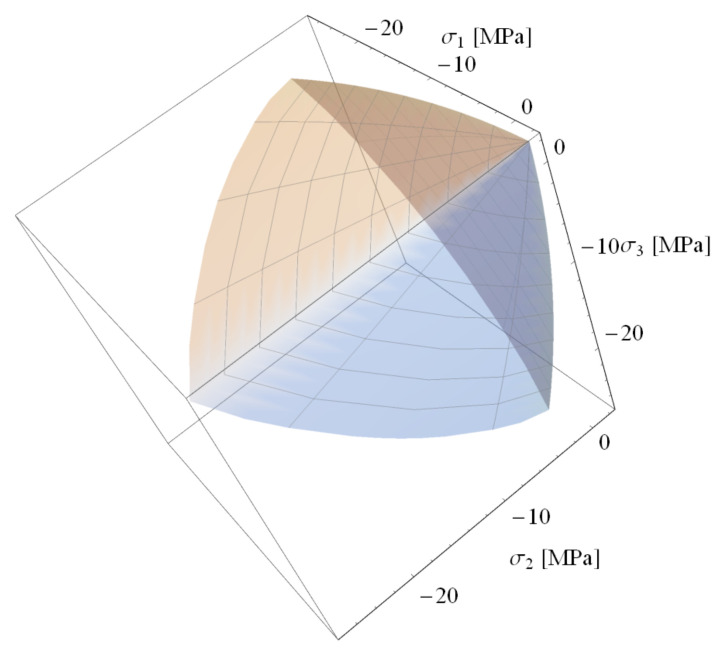
Three-dimensional view of the JP surface.

There are five parameters in the formulas describing the boundary surface of concrete: α, β, $C_{0}$, $C_{1}$, and $C_{2}$, which can be calibrated based on the following empirically determined concrete strengths: uniaxial and triaxial tensile strength, uniaxial compressive strength, and biaxial compressive strength (for two principal stress ratios $\frac{\sigma_{1}}{\sigma_{2}}=1$ and $\frac{\sigma_{1}}{\sigma_{2}}=2$). Formulas for calculating all parameters of the *PJ* surface based on the above-mentioned strengths and comparison of surface shape with the better-known William-Warnke [40] surface can be found in Reference [41]. A three-dimensional view of the *PJ* surface is shown in Figure 5.

### 2.3. Smeared Crack Model and Crushing

The implemented smeared crack model is based on the Rashid concept [42], which later was developed by Vidosa et al. [43], Kotsovos and Spiliopoulos [44], Spiliopoulos and Lykidis [16], Markou and Papadrakakis [18], Mourlas et al. [20,21], and Engen et al. [22]. In this approach, the modification of stiffness properties and stress values is introduced in Gauss points, at which the stress state reached the ultimate surface. After crack occurrence, the greatest principal stress is set to a value near zero and a constitutive matrix with residual stiffness perpendicular to the crack surface is used in the following incremental-iterative process.

**Figure 6:**
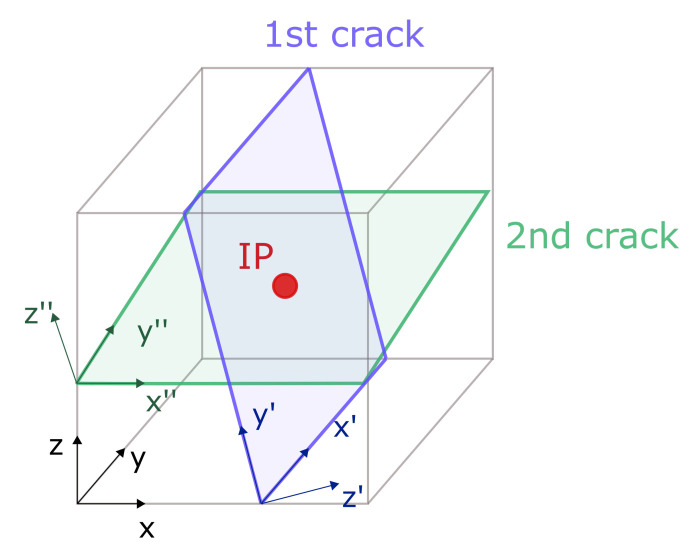
Local coordinates systems associated with cracks.

In the proposed model, there is a possibility to open up to two non-orthogonal cracks at each integration point, which is schematically shown in Figure 6. The second crack can open if the angle between normals in the first and second cracks exceeds the threshold value.

In non-linear FEM codes, one has to define the incremental relation:

(12) $$d\sigma =D_{t}\;d\epsilon \;,$$

where $D_{t}$—tangent constitutive matrix, and dσ, dϵ—vectors of stress and strains increments (in the Voigt notation):

(13) $$d\sigma ={\left[\begin{array}{cccccc} d\sigma_{xx} & d\sigma_{yy} & d\sigma_{zz} & d\sigma_{xy} & d\sigma_{xz} & d\sigma_{yz} \end{array}\right]}^{T},$$

(14) $$d\epsilon ={\left[\begin{array}{cccccc} d\epsilon_{xx} & d\epsilon_{yy} & d\epsilon_{zz} & d\gamma_{xy} & d\gamma_{xz} & d\gamma_{yz} \end{array}\right]}^{T}.$$

As for isotropic material, in the case of an “uncracked” integration point, the tangent material matrix is used to update the stress vector:

(15) $$D_{t}=D=\left[\begin{array}{cccccc} 2G_{t}+\lambda_{t} & \lambda_{t} & \lambda_{t} & 0 & 0 & 0\\ \lambda_{t} & 2G_{t}+\lambda_{t} & \lambda_{t} & 0 & 0 & 0\\ \lambda_{t} & \lambda_{t} & 2G_{t}+\lambda_{t} & 0 & 0 & 0\\ 0 & 0 & 0 & G_{t} & 0 & 0\\ 0 & 0 & 0 & 0 & G_{t} & 0\\ 0 & 0 & 0 & 0 & 0 & G_{t} \end{array}\right],$$

where $\lambda_{t}=K_{t}-\frac{2}{3}\;G_{t}$—Lame constant, and $K_{t}$, $G_{t}$ are calculated according to Equation (3) for the initial value of the octahedral shear strain $\gamma_{oct}$.

When the value of $F(\sigma_{oct},\tau_{oct},\varphi )$ becomes positive for the first time, and at least one principal stress is positive, the crack initiation algorithm is called. The maximum principal value is set to the residual value, and the stress vector is transformed to a global coordinate system with the following formulas:

(16) $$\sigma =T\;{\left[\begin{array}{cccccc} \sigma_{1} & \sigma_{2} & \sigma_{3}\to r_{\sigma }f_{t} & 0 & 0 & 0 \end{array}\right]}^{T},$$

where: $r_{\sigma }$—the residual stress coefficient ($0\leq r_{\sigma }\leq 1$), and T—the transformation matrix (calculated according to Reference [17]). In contrast to the aforementioned papers (e.g., References [20,21,22]), the stress in cracks is not set equal to zero due to different convergence criteria for the Newton scheme. During tests, it was found that the best stability of the incremental process is observed for $r_{\sigma }\approx 0.2$. A physical interpretation of this parameter could be the measure of tension softening, which characterises plain concrete. During the following incremental-iterative process, the tangent constitutive matrix is used (in global coordinates):

(17) $$D_{t}=T\;D^{\prime }\;T^{T}=T\;\left[\begin{array}{cccccc} 2G_{t}+\lambda_{t} & \lambda_{t} & 0 & 0 & 0 & 0\\ \lambda_{t} & 2G_{t}+\lambda_{t} & 0 & 0 & 0 & 0\\ 0 & 0 & \beta_{n}\left(2G_{t}+\lambda_{t}\right) & 0 & 0 & 0\\ 0 & 0 & 0 & G_{t} & 0 & 0\\ 0 & 0 & 0 & 0 & \beta_{s}G_{t} & 0\\ 0 & 0 & 0 & 0 & 0 & \beta_{s}G_{t} \end{array}\right]\;T^{T},$$

where: $\beta_{n}$—normal retention factor (a value of $10^{-4}$ was adopted as in References [22,29]), and $\beta_{s}$—shear retention factor (a value of 0.1 was adopted as in References [17,22]). Retention factors are introduced mainly to maintain the numerical stability of the incremental process [17,45]. It should be noted that the Poisson effect in the crack plane disappears after crack formation (the appropriate non-diagonal components are set to 0).

The second crack opens if three conditions are met simultaneously: function $F(\sigma_{oct},\tau_{oct},\varphi )$ becomes positive for the second time, maximum principal stress $\sigma_{3}\geq r_{\sigma }f_{t}$, and the angle between normal to the existing and potential crack is greater than 45°. If the angle is less than 45°, all components of the stress vector are scaled by the same coefficient η:

(18) $$\eta =\frac{\sqrt{4C_{0}\;C_{2}\;\tau_{oct}^{2}+{\left(\sigma_{oct}+C_{1}\;P\;\tau_{oct}\right)}^{2}}-\sigma_{oct}-C_{1}\;P\;\tau_{oct}}{2C_{2}\;\tau_{oct}^{2}}.$$

Equation (18) for coefficient η was obtained by solving F(η σ)=0. Hence, the stress vector is mapped on the *PJ* surface. A similar solution was adopted by Engen [22]. The value of the threshold angle was selected based on References [22,46,47]. For smaller values of this angle, the algorithm can suffer from spurious cracking, and, for larger values, the stress state can go beyond the failure criterion (this is a well-known drawback of the fixed orthogonal cracks concept [47]).

The second crack formation is connected with the setting of two normal stresses in a new local coordinate system to residual values and the following modification of the tangent constitutive matrix:

(19) $$D_{t}=T\;D^{\prime \prime }\;T^{T}=T\;\left[\begin{array}{cccccc} 2G_{t}+\lambda_{t} & 0 & 0 & 0 & 0 & 0\\ 0 & \beta_{n}\left(2G_{t}+\lambda_{t}\right) & 0 & 0 & 0 & 0\\ 0 & 0 & \beta_{n}\left(2G_{t}+\lambda_{t}\right) & 0 & 0 & 0\\ 0 & 0 & 0 & \beta_{s}G_{t} & 0 & 0\\ 0 & 0 & 0 & 0 & \beta_{s}G_{t} & 0\\ 0 & 0 & 0 & 0 & 0 & \beta_{s}G_{t} \end{array}\right]\;T^{T}.$$

If the stress vector reaches the ultimate surface for the third time with all principal stresses tensile, the algorithm of scaling stresses is called. Hence, the third crack is modelled in a ductile manner. It was found that such a solution is a good compromise between the stability of the incremental process and the physical background, since the state of triaxial tension is rather rare in real building structures and Gauss points with triple cracks often lead to convergence problems. Hence, for example, in ANSYS code in the SOLID65 element, dedicated to the NLFEA of RC structures, users can deactivate the third crack [48]. In Reference [18], the possibility of opening the third crack is not included in the algorithm.

If the stress vector reaches the ultimate surface, and all principal stresses are compressive or of zero value, the algorithm for concrete crushing is called. The softening phase starts in the direction of minimum principal stress, whereas normal stresses perpendicular to the minimum principal stress are set to zero. The constitutive matrix is modified in the following manner:

(20) $$D_{t}=T\;D^{\prime \prime \prime }\;T^{T}=T\;\left[\begin{array}{cccccc} \beta_{c}E_{0} & 0 & 0 & 0 & 0 & 0\\ 0 & \beta_{n}\left(2G_{t}+\lambda_{t}\right) & 0 & 0 & 0 & 0\\ 0 & 0 & \beta_{n}\left(2G_{t}+\lambda_{t}\right) & 0 & 0 & 0\\ 0 & 0 & 0 & \beta_{s}G_{t} & 0 & 0\\ 0 & 0 & 0 & 0 & \beta_{s}G_{t} & 0\\ 0 & 0 & 0 & 0 & 0 & \beta_{s}G_{t} \end{array}\right]\;T^{T},$$

where: $\beta_{c}$—parameter that controls softening rate. This parameter can be related to the fracture energy in compression:

(21) $$\beta_{c}=-\frac{L_{e}}{b}\frac{f_{c}^{2}}{2G_{c}E_{0}},$$

where: $L_{e}=\min\left(150\;\mathrm{mm},h_{e}\right)$; $h_{e}$—characteristic element length (the lower bound was introduced, since models consisting of small elements predict very ductile behaviour of structures [49]), $G_{c}$—fracture energy for compression, *b*—parameter, which allows for the increased ductility of a reinforced compressive zone to be taken into account (b=1 for an unreinforced compressive zone and b=10 for a reinforced compressive zone), and $E_{0}$—initial Young modulus. Fracture energy in compression can be calculated according to ($f_{c}$ substituted in MPa) [50]:

(22) $$G_{c}\approx 3650÷7300f_{c}^{0.18}.$$

**Figure 7:**
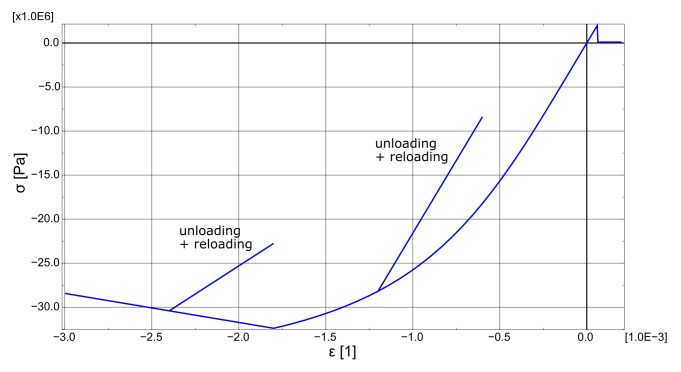
Behaviour of the proposed model in the uniaxial stress state.

**Figure 8:**
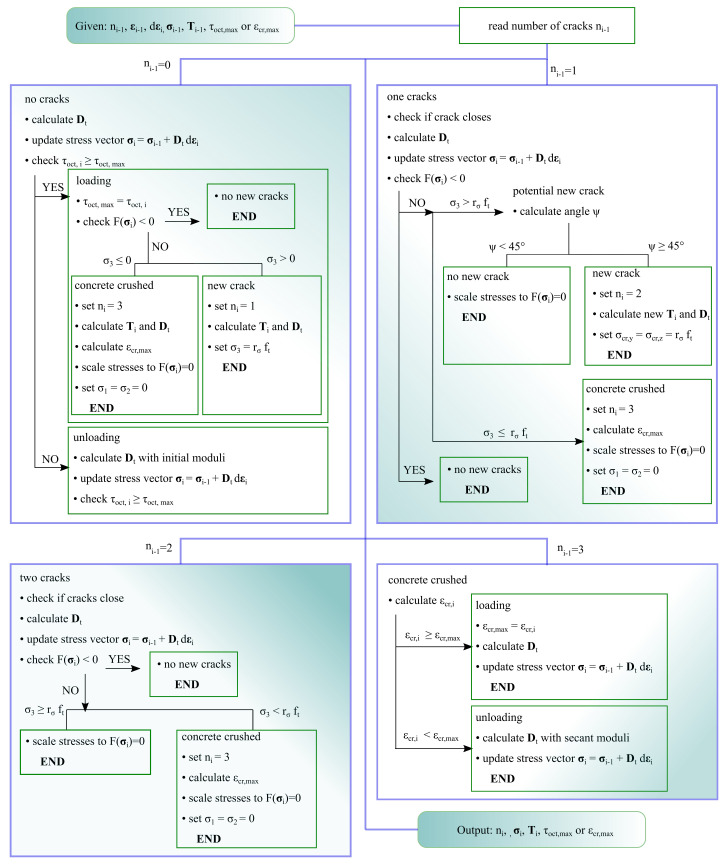
Flowchart with the implemented algorithm for the material model of concrete. Subscript i−1 represents the value for the previous iteration, while i value for the present iteration.

As an indicator for loading and unloading in uncracked Gauss points, octahedral shear stress $\tau_{oct}$ was selected. If $\tau_{oct,i}$ for the current increment is bigger than for the previous $\tau_{oct,i-1}$, then $\tau_{oct,max}=\tau_{oct,i}$. The condition $\tau_{oct,i}<\tau_{oct,max}$ indicates unloading or reloading. In this case, the initial values of modules $K_{0}$, $G_{0}$ are used in matrix $D_{t}$ calculated according to Equation (15). The algorithm is similar to other algorithms described in the literature [22,29,44]. In “crushed” Gauss points, at which strain softening occurs, the local normal strain is used as an indicator of unloading/reloading. In unloading and reloading at the strain-softening stage, the secant Young modulus is used as in Reference [51]. Moreover, the proposed model includes a crack closure algorithm similar to the one described in Reference [44]. Cracks close when the strains normal to the crack turn their sign from positive to negative. Then, the stiffness matrix for uncracked material or material with a single crack is used, depending on the number of opened cracks. The behaviour of the proposed model in uniaxial stress state with unloading and reloading branches is depicted in Figure 7, whereas the flowchart which shows the order of operations execution can be found in Figure 8.

### 2.4. Generalised Constitutive Model of Steel

The generalised constitutive model for steel was derived from provisions of the fib Model Code 2010 [31] in a similar way as in Reference [24]. In this monograph, it was assumed that concrete carries no tensile stresses and the entire stiffness of the tensile zone is included via a constitutive model of steel. In the present model, TS is activated after crack occurrence, since concrete earlier behaves like an isotropic continuum.

**Figure 9:**
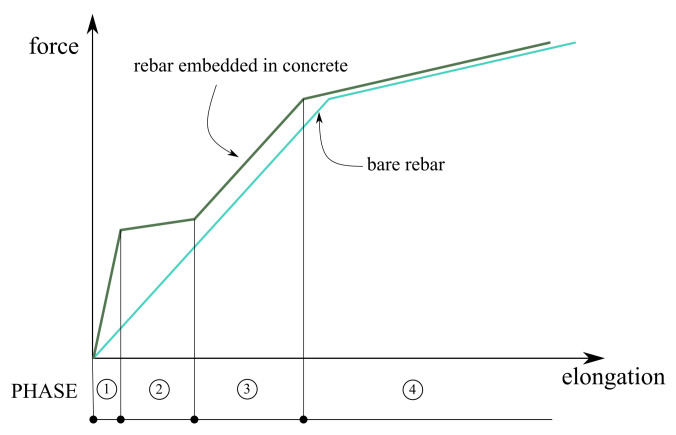
Typical force—elongation curve for reinforcement bar embedded in concrete. 1—elastic stage, 2—crack formation, 3—stabilised cracks, 4—plastic stage.

According to the fib Model Code 2010 [31], we can distinguish four phases in the tension of the reinforcement bar embedded in concrete (see Figure 9):

- Phase 1—elastic stage, in which concrete and steel behave in a linear manner and have equal strains;
- Phase 2—crack formation, which begins when stresses in concrete cover reach tensile strength;
- Phase 3—stabilised cracking; and
- Phase 4—yielding of steel, in which the TS effect vanishes due to the degradation of the bonding properties between reinforcing steel and concrete.

**Figure 10:**
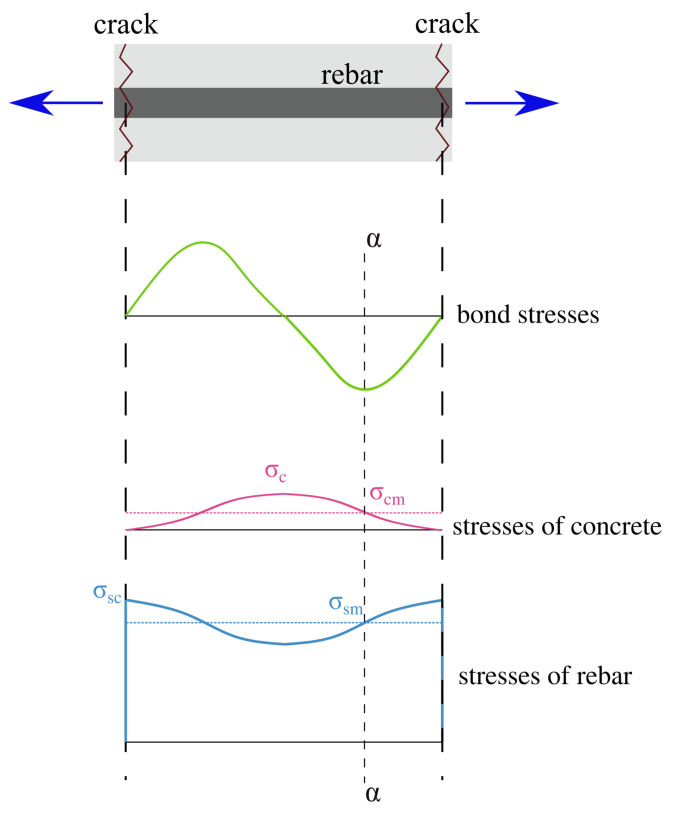
Stress distributions in reinforcement bar in concrete cover.

The relationship that applies in phase 3 can be derived on the basis of an equilibrium equation of the reinforcement bar in tension. Stress distribution in such a simple structure is shown in Figure 10. The following relationship, which links steel stress in crack ($\sigma_{sc}$) and mean strain between cracks ($\epsilon_{sm}$) after composing the equilibrium equation for the segment between α—α section and the crack section can be easily derived from:

(23) $$\sigma_{sc}=E_{s}\;\epsilon_{sm}+\beta_{t}\frac{f_{ct}}{\rho_{eff}}\;,$$

where: $E_{s}$—Young modulus of reinforcing steel, $\epsilon_{sm}$—mean strain of the reinforcement bar between cracks, $\rho_{eff}$—effective reinforcement ratio, and $\beta_{t}$—parameter, which characterises the quality of the bond (for typical deformed bars: $0.4\leq \beta_{t}\leq 0.6$).

The effective reinforcement ratio is calculated according to the following formulas:

(24) $$\rho_{eff}=\frac{A_{s}}{A_{c,eff}}\;,$$

where: $A_{s}$—cross-sectional area of reinforcement, and $A_{c,eff}$—effective tension area, which can be calculated according to Section 7.6.4.4 in [31].

After some rearrangements, the generalised constitutive model of reinforcing steel has the following multi-linear form:

(25) $$\sigma_{s}\left(\epsilon_{s}\right)=\left\{\begin{array}{cc} E_{s}\epsilon_{s} & \mathrm{for}\;\epsilon_{s}<\frac{f_{t}}{E_{c}}\\ E_{s}\;\epsilon_{s}\left(1-\frac{\beta_{t}\;E_{c}}{a}\right)+\frac{f_{t}\;E_{s}}{a} & \mathrm{for}\;\frac{f_{t}}{E_{c}}\leq \epsilon_{s}<f_{t}\left(\frac{\alpha -\beta_{t}}{E_{s}\;\rho_{eff}}+\frac{\alpha }{E_{c}}\right)\\ E_{s}\;\epsilon_{s}+\beta_{t}\frac{f_{t}}{\rho_{eff}} & \mathrm{for}\;f_{t}\left(\frac{\alpha -\beta_{t}}{E_{s}\;\rho_{eff}}+\frac{\alpha }{E_{c}}\right)\leq \epsilon_{s}<\frac{1}{E_{s}}\left(f_{y}-\beta \frac{f_{t}}{\rho_{eff}}\right)\\ f_{y}+E_{t}\left(\epsilon_{s}-\epsilon_{y}\right) & \mathrm{for}\;\epsilon_{s}\geq \frac{1}{E_{s}}\left(f_{y}-\beta \frac{f_{t}}{\rho_{eff}}\right) \end{array}\right.,$$

where: α—parameter that indicates the end of the crack formation phase (1.0≤α≤1.3), $f_{y}$—yield stress, and $E_{t}$—hardening modulus, and $a=E_{c}\;\left(\beta_{t}-\alpha \right)+\rho_{eff}\;E_{s}\;\left(\alpha -1\right)$.

**Figure 11:**
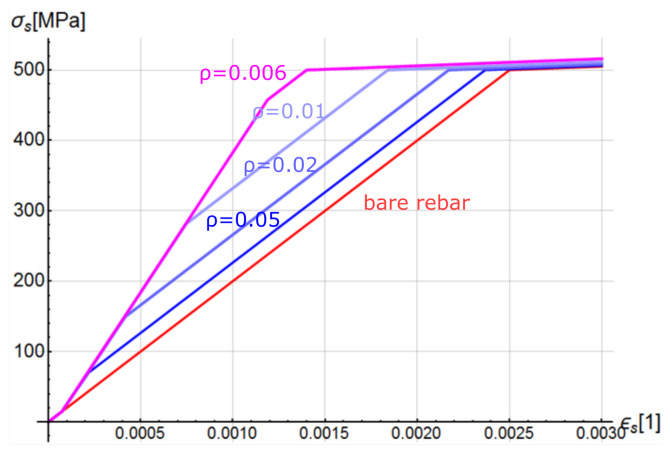
The σ – ϵ curves for the proposed generalised constitutive model of reinforcing steel—parametric study for different reinforcement ratios $\rho_{eff}$.

The graphs of function $\sigma_{s}\left(\epsilon_{s}\right)$ are shown in Figure 11. A parametric study of the effective reinforcement ratio and a comparison with the bare reinforcement bar were performed. Based on an analysis of the chart, as the effective reinforcement ratio decreases, TS has greater influence on structure deformability. This observation is consistent with the results of experimental studies [25]. On the other hand, the latest experimental research performed on RC tension members revealed that taking into shrinkage strains reduces influence of reinforcement ratio [52].

### 2.5. Implementation, Finite Elements, Solution Method, Convergence Criteria

The described material models were implemented into the Abaqus code (Abaqus 2020, License: SIMULIA Academic Abaqus Research Suite for Building Research Institute in Warsaw, Poland) via the user material procedure *UMAT* for solver Abaqus/Standard written in the FORTRAN language [27]. The *UMAT* procedure is called in every Gauss point and has to update: the stress vector, the internal variables and the material stiffness matrix. User procedures are common in scientific research [32,51,53], since they enable the user to focus on a specific phenomenon and the rest of the numerical model can be covered with algorithms available in the FEM code.

Based on provisions formulated in Reference [22], 8-noded solid elements C3D8 [27] were used to model the concrete domain. Within these elements, selective reduced integration was used, as described in Reference [54]. This eliminates volumetric locking, but shear locking characteristic for low-order elements remains. This has significant influence for rather thin structural elements, which are rarely made of reinforced concrete. It is well known that the concept of full reduced integration reduces shear locking, but the stiffness matrix of such elements has additional zero-energy modes, which in combination with the smeared cracks concept (additional eigenvalues of the element stiffness matrix close to zero) results in problems with numerical stability [17,45,46]. Furthermore, based on a survey of the literature, Engen et al. [22] stated that low-order elements have better performance in the NLFEA of RC structures than higher order elements, since the latter can lead to artificial straining and premature cracking. Besides, the application of low-order elements enables to obtain reasonable dimensions of the global stiffness matrix and, consequently, faster solution of each equilibrium iteration. Simple truss elements T3D2 were used to model reinforcement [27]. The elements were connected together with the *embedded* option [18,27], for which solid and truss elements nodes do not have to be in the same location. Hence, regular and coarse meshes can be used in the analysis. In terms of mesh size, the constitutive models that predict decrease in stresses with increasing strains could suffer from pathological mesh dependency. Conversely, this phenomenon appears mostly in plain concrete and very fine meshes, which are not desirable for the NLFEA of large-scale RC structures. Based on the conclusions of Reference [49], the following limit values for element size are recommended:

- mesh size should be larger than approximately 1.5 the aggregate size (approximately 50 mm),
- mesh size should be smaller than typical crack spacing (approximately 150 mm).

As demonstrated in the literature [16,18,22], for such element dimensions the mesh dependency for brittle models is negligible. For finer meshes, the convergence problems can occur, as well as FE models may underestimate the structural element stiffness [55].

The quasi-Newton method (BFGS — Broyden–Fletcher–Goldfarb–Shanno — variant) was used to find the equilibrium paths of the structures, but the full Newton method was also used for comparison purposes. The basis of the BFGS algorithm application to non-linear mechanics was described in Reference [56]. The performance of this method in problems of non-linear mechanics is demonstrated with an example of an RC beam in Reference [57]. Moreover, an important observation is made there, that fewer load increments are not equivalent to shorter calculation time due to the larger number of equilibrium iterations needed. Based on these provisions, the automatic control of load increments length available in Abaqus was used, which proves to cooperate effectively with the proposed material models.

In the Abaqus code, the following convergence criterion is used for the iterative process [27]:

(26) $$\max\left(F^{N}\right)\;<\;R\;\tilde{F}\;,$$

where: $\max(F^{N})$—maximum component of unbalanced force vector, *R*—convergence tolerance for force (assumed as 0.02), and $\tilde{F}$—average value of nodal force vector. Additionally, the condition must be met that the displacement correction for a given load increment is not too large:

(27) $$\max\left(c^{N}\right)\;<\;C\;\max\left(\Delta u\right)\;,$$

where: $\max(c^{N})$—the largest correction of the displacement vector resulting from the iteration or its value predicted for the next iteration, C—convergence tolerance for vector of displacement correction (assumed as 0.01—default value), and max(Δu)—the maximum component of displacement increment vector for a given load increment.

The script was coded in Python in order to visualise the crack patterns for different load levels. This creates an additional field in the output database [27] with vectors in the cracks plane. Only the final crack direction is visualised due to limitations of the program’s post-processor.

## 3. Verification and Validation of the Proposed Strategy

Every new solution strategy, prior to industrial or research implementation, should be verified and validated (V&V) [22,58]. In general, the difference between validation and verification is accurately expressed by the two following statements by Roarche [59] (cited in Kwasniewski and Bojanowski [58] and Engen et al. [22], respectively): “Verification deals with mathematics, validation deals with physics”. Verification answers the question “are we solving the equations right?”, while validation answers the question “are we solving the right equations?”. According to Reference [22], in the case of the NLFEA of RC structures, verification should cover a comparison of the results obtained with: different FE meshes, load step lengths, and different incremental-iterative procedures, while validation is based on the simulation of benchmark, reliable experiments. The validation procedure proves model objectivity and can prove model generality if one chooses the right set of benchmarks. In the present paper, V&V were performed jointly (as in References [18,21]) and were divided into four case studies (CS). Additional results of benchmark analysis obtained with the proposed models can be found in Reference [60].

### 3.1. CS1 Numerical Willam’s Test

The main non-linear effect in the RC structures is cracking. Hence, the proper reproduction of this phenomenon is crucial for the correct prediction of the load capacity and stiffness of the structure. In order to assess different concepts of including the cracking phenomenon into FE models, the Willam’s test was proposed [61]. The results of this test for material models available in the Diana code can be found in Reference [47], whereas comprehensive study on among others models available in the Abaqus code is summarised in Reference [62]. The test consists of two phases:

- phase 1—uniaxial tension up to the crack formation, components of the strain increments vector in the Cartesian coordinate system have the following ratio: $\Delta \epsilon_{xx}:\Delta \epsilon_{yy}:\Delta \gamma_{xy}=1:\nu :0$;
- phase 2—biaxial tension and shear, components of the strain increments vector in the Cartesian coordinate system have the following ratio: $\Delta \epsilon_{xx}:\Delta \epsilon_{yy}:\Delta \gamma_{xy}=0.5:0.75:1$.

**Figure 12:**
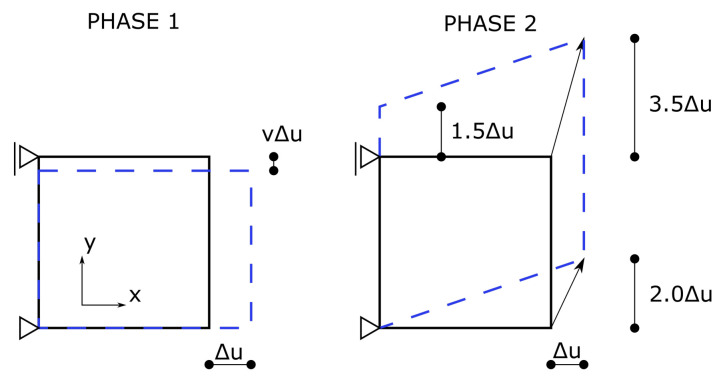
The Willam’s test—boundary conditions and coordinate system.

The boundary conditions which correspond to these two phases are shown in Figure 12. The test is passed if two conditions resulting from physical premises are met:

- during the test, the larger principal stress does not exceed the tensile strength;
- at the end of phase 2 all stresses tend to zero.

**Figure 13:**
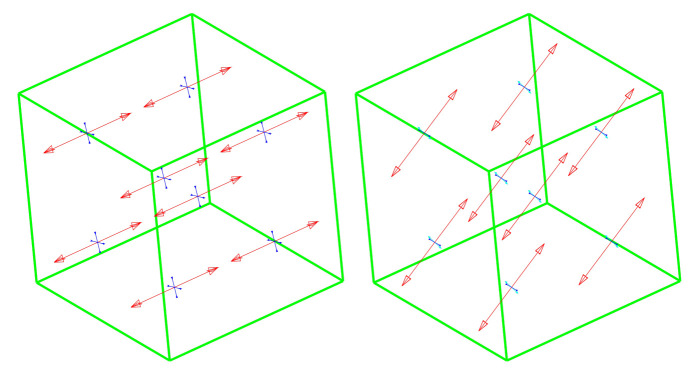
Willam’s test—principal directions at the end of phase (from left): 1 and 2.

The Willam test is used to assess the correctness of the smeared cracks models in loading conditions, where the principal directions of the strain vector change. These directions, during phase 1 and at the end of phase 2, are shown in Figure 13.

**Figure 14:**
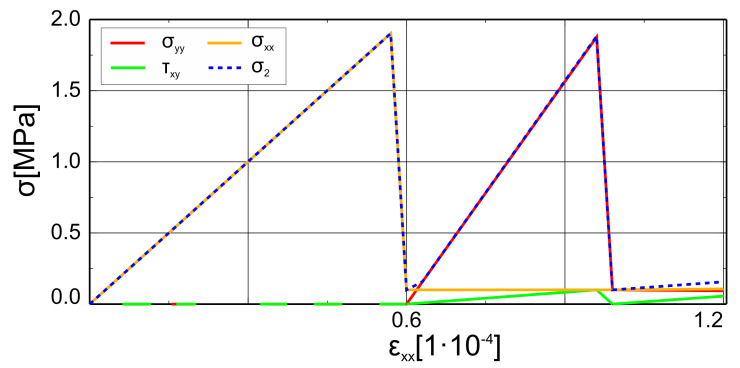
Willam’s test—change of stress values in the two phases.

The adopted parameters of the proposed model can be found in Table 1. The results of Willam’s test as functions of stress values depending on the value of strain $\epsilon_{xx}$ are shown in Figure 14. The test can be considered as conditionally passed: the first condition was met, but stress values at the end of phase 2 rose. This increase, however, is very slow due to the small value of the assumed shear retention factor—0.1. The test results could be easily improved by assuming zero shear retention factor. The shear retention factor, however, is crucial in terms of the cracked element stiffness matrix, as mentioned before in Section 2.3. Therefore, the proposed model is a good compromise between the physical behaviour of brittle material and numerical efficiency.

### 3.2. CS2 Beam in Bending

**Figure 15:**
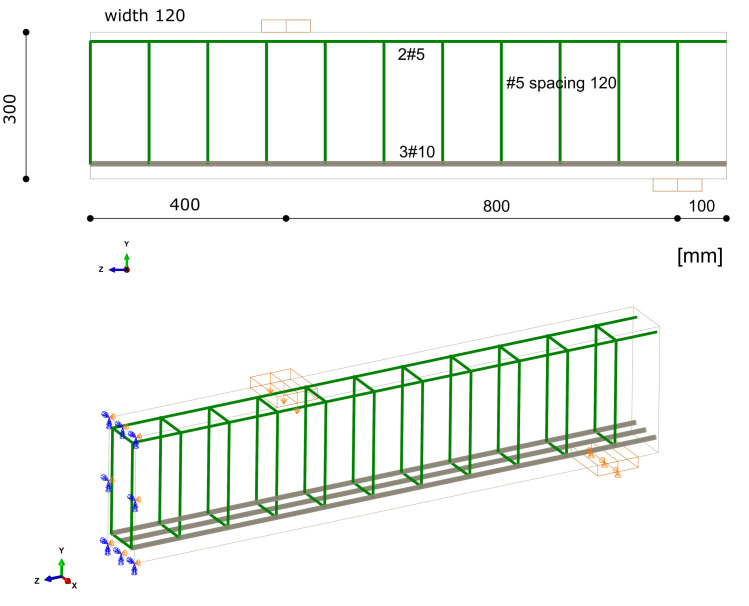
Beam failure in bending—dimensions and boundary conditions assumed in the analysis.

**Figure 16:**
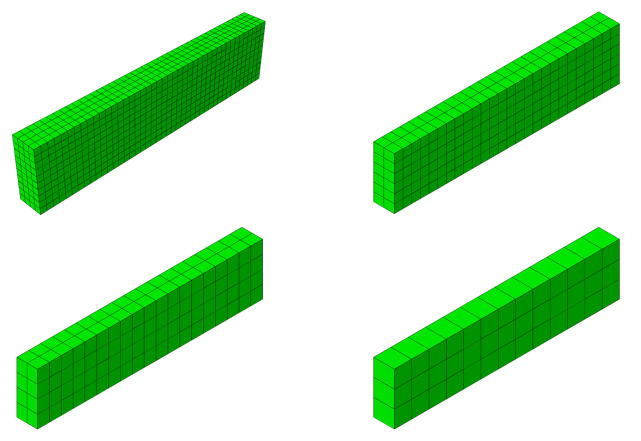
Meshes assumed in case study 2 (CS2).

Case study 2 concerns the failure of a beam in bending, which was experimentally and numerically investigated by Alvarez [63]. Its behaviour was recently simulated by Gomes [64]. The beam was tested in a four-point bending scheme. The geometrical features and boundary conditions assumed in the numerical analysis are depicted in Figure 15. Nodes on surfaces of steel plates were tied with nodes of concrete in their vicinity. Due to the symmetry of the beam and the test setup, a half of the beam was modelled. Displacement control was used in the analysis. Four analysed meshes (element side dimensions: 30, 50, 70, and 100 mm) are shown in Figure 16. The parameters of the proposed models adopted in the analysis are summarised in Table 1.

**Figure 17:**
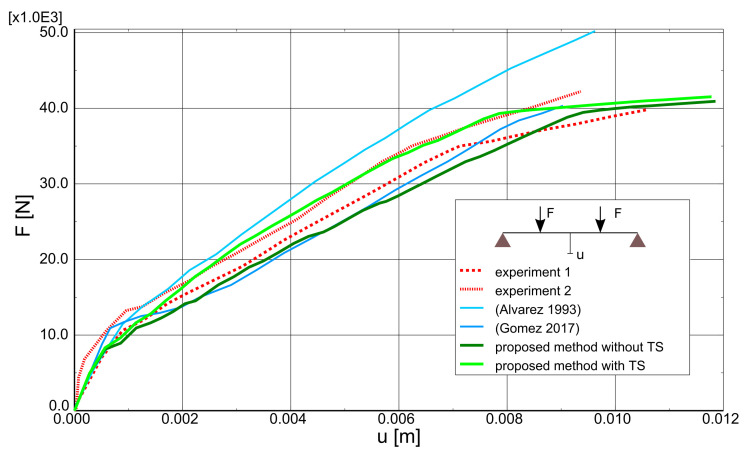
Beam failure in bending—influence of tension stiffening (TS).

A comparison of test and analytical charts showing the relationship between the total force acting on the beam (*F*) and the deflection of its midpoint (*u*) is given in Figure 17. The *F*-*u* curves obtained with the present method were compared with the results of Alvares [63] and Gomes [64]. The first used Mazars’ damage model [65] for concrete with strain-softening in tension and compression. Overestimation of beam load capacity occurred in this model due to the elastic model of reinforcing steel. Overestimation of beam stiffness, however, was visible well before the yielding of the steel. Hence, modelling TS as a descending branch in the concrete constitutive model may lead to an exaggeration of this phenomenon. Gomes [64] used the SOLID65 element available in ANSYS code, in which an elasto-brittle-plastic model of concrete is implemented. The smeared cracks model follows the fixed-orthogonal cracks approach [48]. The results of this analysis are quite similar to the results obtained with the proposed method neglecting tension stiffening, which proves the correctness of the smeared cracks algorithm implemented by the author. Moreover, these results show a slightly underestimated stiffness. The best agreement with experimental results was obtained for the proposed model including TS via modification of the constitutive model of reinforcing steel.

**Figure 18:**
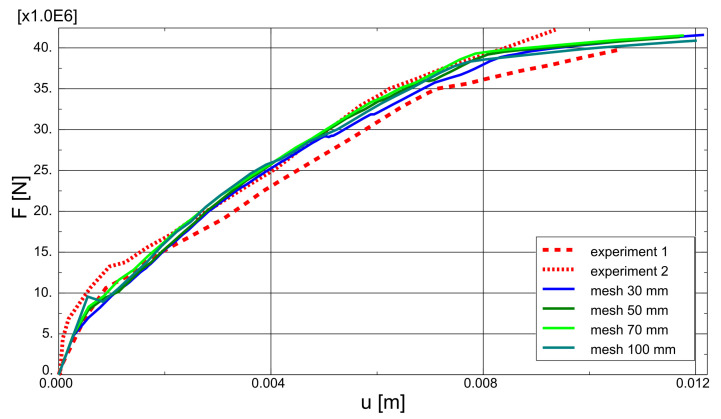
Beam failure in bending—mesh dependency study.

**Figure 19:**
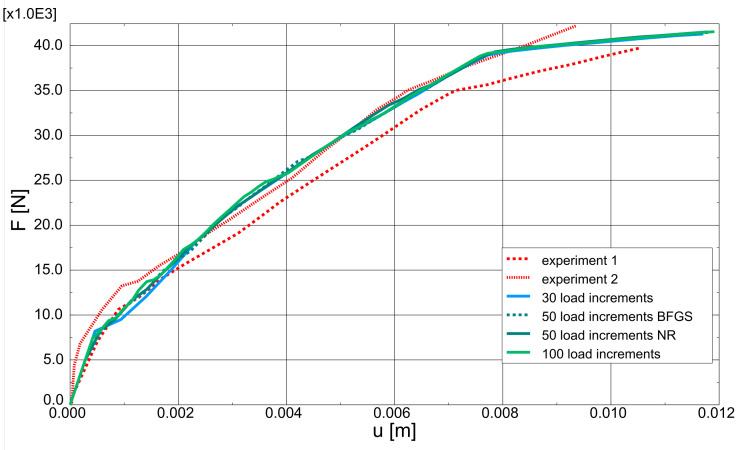
Beam failure in bending—influence of Newton-Raphson parameters.

**Figure 20:**
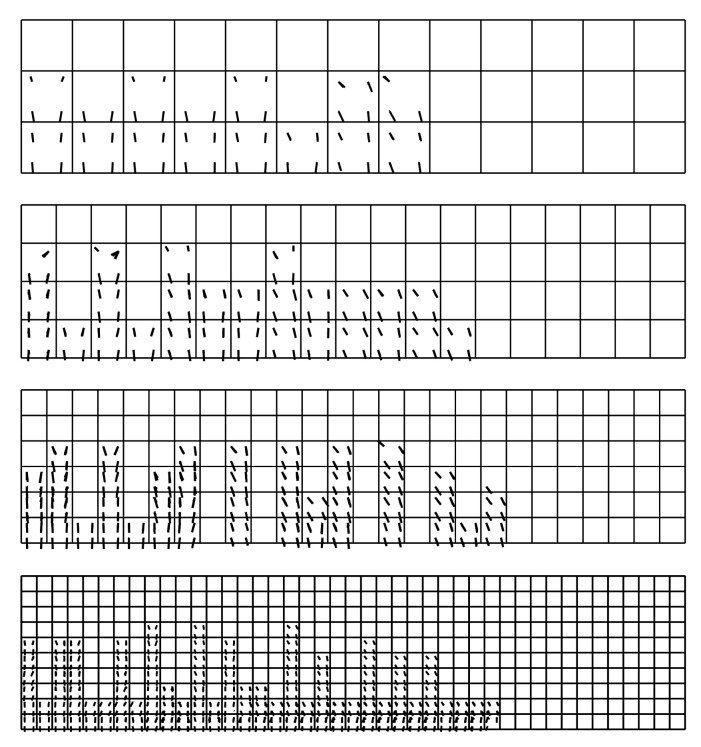
Beam failure in bending—crack patterns for different meshes—deflection: 2.7 mm.

As part of the current CS, the proposed model was also verified by: performing mesh dependency studies (the results can be found in Figure 18), performing analysis with different load increments (30, 50, and 100) and two Newton schemes (default BFGS and full Newton-Raphson; see Figure 19). Crack patters for analysed meshes can be found in Figure 20. The results of the performed tests show that the proposed model is not very sensitive for mesh and load increment length change, so it can be considered objective.

### 3.3. CS3 Beam without Stirrups in Shear

Case study 3 concerns the failure of beam OA-1 without stirrups in shear, which was one of the series tested by Bresler and Scordelis [66]. This is a classic benchmark for validating new NLFEA strategies for RC structures [17,18,53,67,68]. Due to the popularity of this experimental data, beams with similar material parameters and dimensions were re-examined by Vecchio and Shim [69]. The results of the re-examination were in good agreement with the original tests, which proves the reliability of the experimental data.

**Figure 21:**
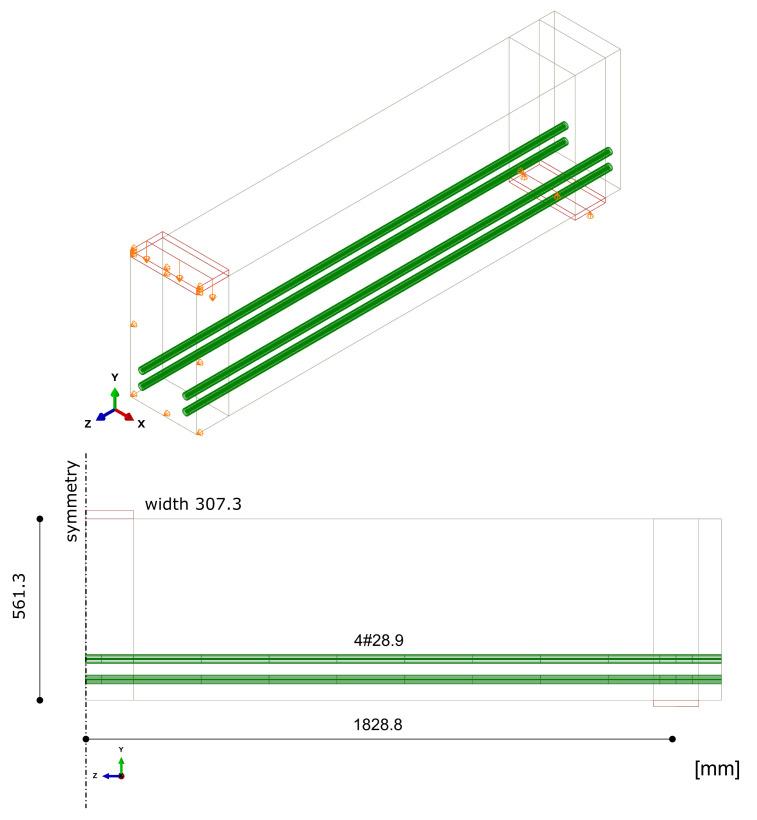
OA-1 beam—dimensions and boundary conditions assumed in the analysis.

**Figure 22:**
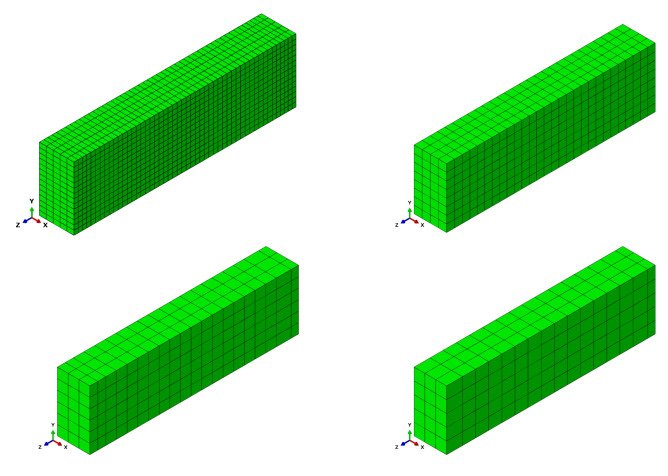
Meshes assumed in CS3 and CS4.

The beam was tested under one-point loading. The geometrical features and boundary conditions assumed in the numerical analysis are depicted in Figure 21. Nodes on surfaces of steel plates were tied with nodes of concrete in their vicinity. Due to the symmetry of the beam and the test setup, a half of the beam was modelled. Displacement control was used in the analysis. Four analysed meshes (element side dimensions: 40, 70, 100, and 120 mm) are shown in Figure 22. The parameters of the proposed models adopted in the analysis are summarised in Table 1.

**Figure 23:**
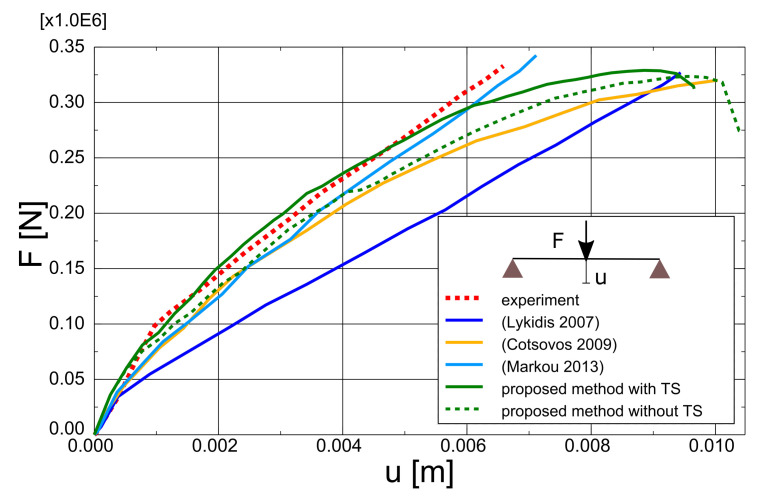
OA-1 beam—influence of TS.

**Figure 24:**
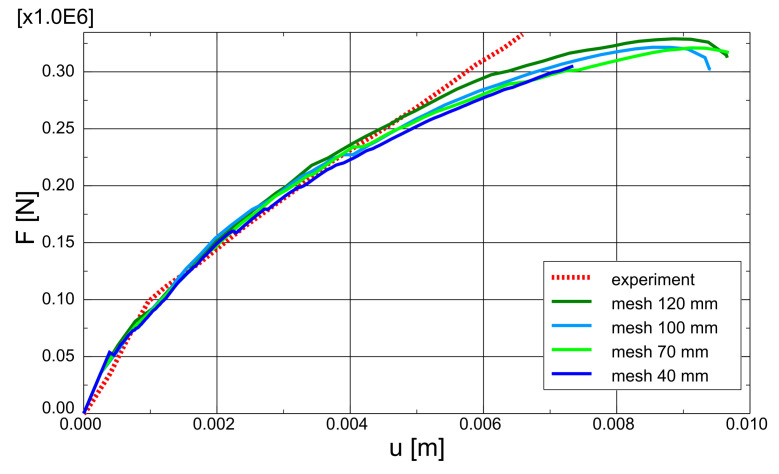
OA-1 beam—mesh dependency study.

**Figure 25:**
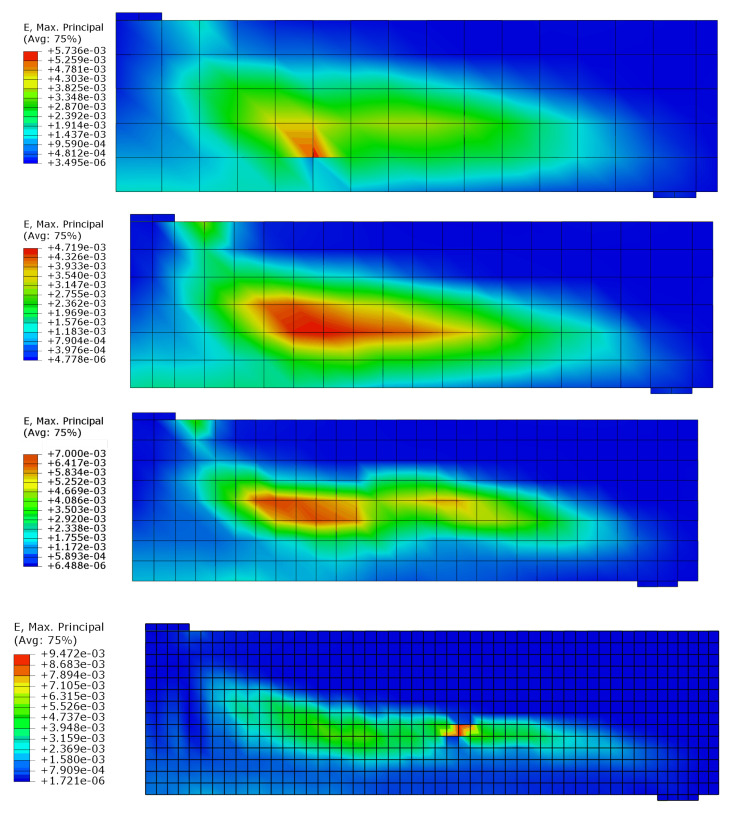
OA-1 beam failure in shear—maps of maximum principal strain.

The test and analytical results in the form of force-deflection (*F* – *u*) graphs are compared in Figure 23. Two variants of the proposed method are shown: with and without TS. The outcomes of other researchers’ FEA are also included in the graph. Lykidis [70] used 27-noded hexagonal elements for concrete, truss elements for rebar, and his analysis neglected TS. Underestimation of beam stiffness is clearly visible in almost the entire load range; however, the load capacity is well predicted. Cotsovos et al. [67] analysed the beam with the SOLID65 element available in ANSYS code, neglecting the TS effect. The results for this model are very similar to the proposed method without TS, which proves the correct implementation of the smeared cracks algorithm in the proposed model. Markou [18] applied 8-noded hexagonal elements for concrete and beam elements for rebar, but his analysis also neglected TS. He obtained very good agreement between the test and analytical results. He deduced that the bending properties of rebars are important for the correct prediction of OA-1 beam stiffness. The method proposed in the present paper, however, was capable of correct beam stiffness prediction for most of the load range. The deflections are overestimated in the vicinity of load capacity—the discrepancy between the experimental and analytical equilibrium paths starts at approximately 80% of the ultimate load. A similar *P* – *u* curve was obtained by Cotsovos et al. [67] using the ANSYS software. The results obtained for different meshes are quite similar (see Figure 24); thus, for larger elements, mesh-dependency is negligible. Maps of maximum principal strains at peak load are shown in Figure 25, which demonstrates the correct prediction of failure mode by the proposed method.

### 3.4. CS4 Beam with Stirrups in Shear

**Figure 26:**
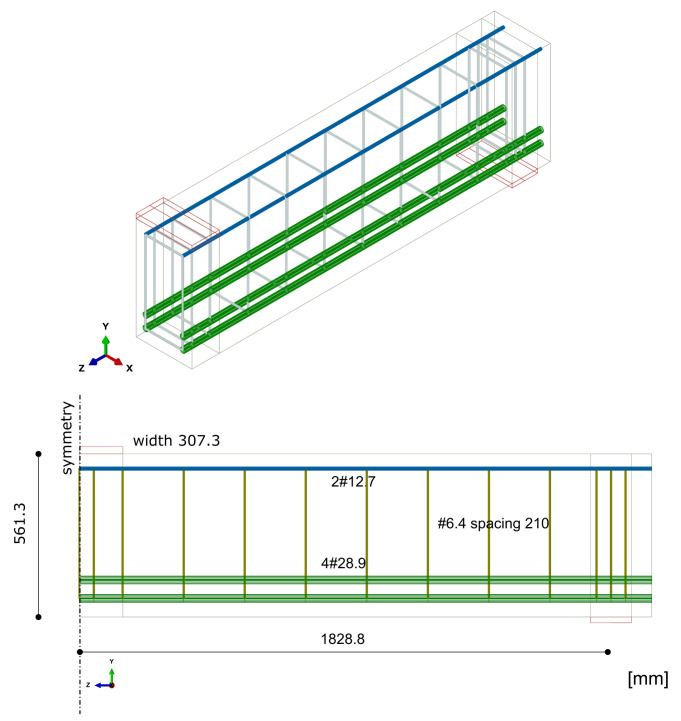
A-1 beam—dimensions and boundary conditions assumed in the analysis.

The final case study also concerns one of the beams tested by Bresler and Scordelis [66]. The A-1 beam had the same dimensions as the OA-1 beam and was tested with the same test stand. The main difference between these beams was the use of stirrups. The geometry of the beam and the boundary conditions assumed in the analysis are shown in Figure 26. Four meshes were analysed, as for CS3 (see Figure 22). Displacement control was used in the analysis, as in the previous CSs. The parameters of the proposed models adopted in the analysis are summarised in Table 1.

**Figure 27:**
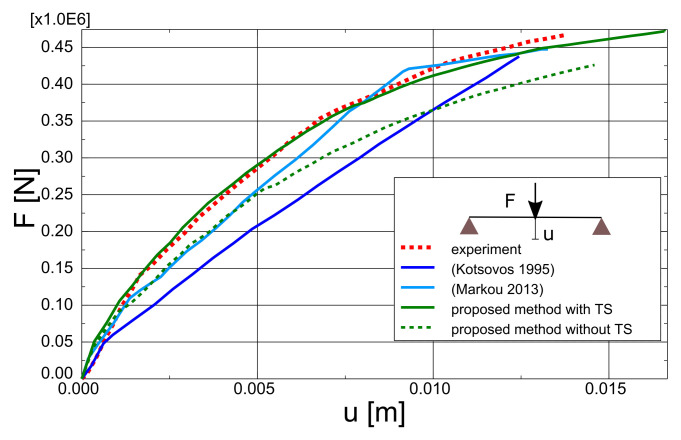
A-1 beam—influence of TS.

**Figure 28:**
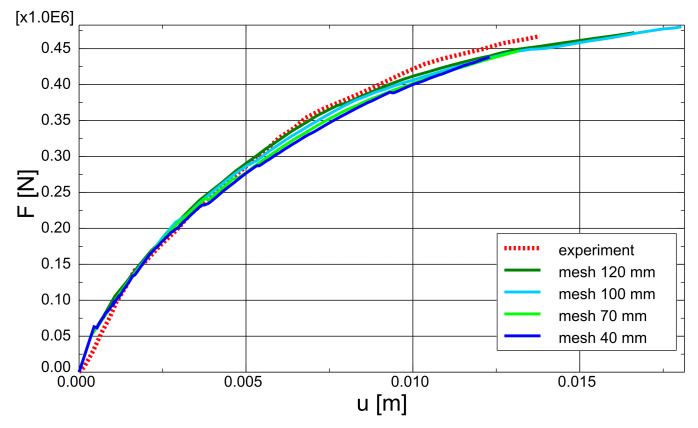
A-1 beam—mesh dependency study.

**Figure 29:**
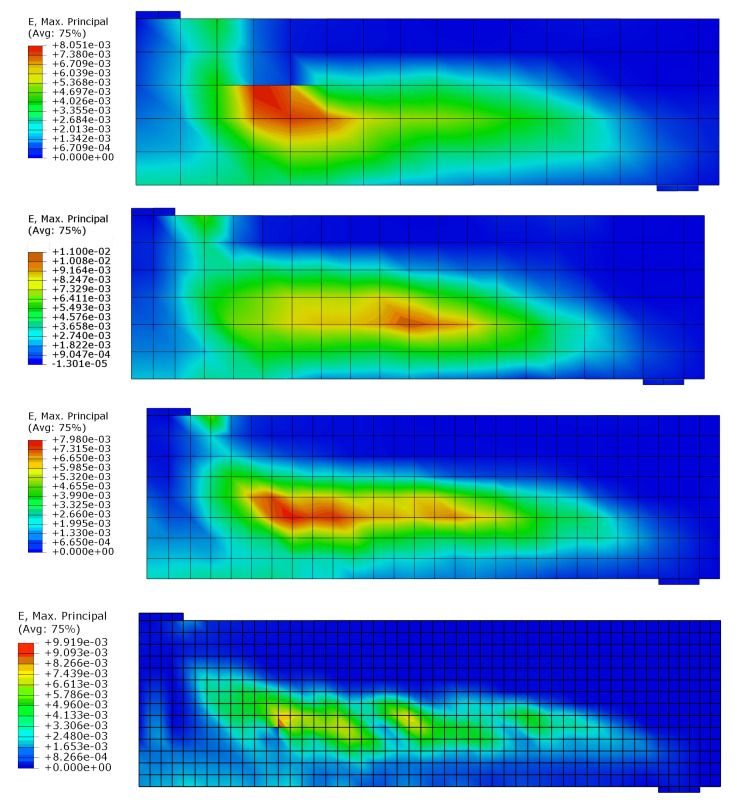
A-1 beam failure in shear—maps of maximum principal strain.

The test and numerical results are compared in Figure 27. The results of analyses taking into account TS and neglecting this phenomenon are shown again, as well as the results obtained by other researchers. Vidosa analysed the A-1 beam with 20-noded hexagonal elements and truss elements [43]. These results were reported in Reference [17]. Markou used 8-noded hexagonal elements for concrete and beam elements for rebar [18]. Both researchers assumed the Kotsovos material model for concrete and neglected TS [17]. Again, better accuracy was obtained by Markou with beam elements. The results of the proposed model, however, are closer to the experimental results in almost the entire load range. The proposed method slightly overestimated the ductility of the A-1 beam. Again, the influence of TS on the deformability of the beam is clearly visible. Results of mesh-dependency studies are shown in Figure 28, whereas maps of maximum principal strains at peak load for different meshes are depicted in Figure 29. The proposed model was capable of the correct prediction of shear failure mode.

## 4. Discussion

Cracking is a crucial non-linear phenomenon in concrete, which determines the mechanical response of RC structures the most. The results presented in CS1 explain the accuracy of the implemented non-orthogonal, fixed smeared cracking algorithm (based on References [18,22,43]) on the material point level. Due to the possibility of opening cracks in non-orthogonal directions, a reasonable choice of threshold angle and the algorithm of scaling stresses is required, while the overall stiffness of the structure is not overestimated, since there are no regions, where the stress state is outside the ultimate surface. Moreover, the orthotropy induced by cracking enables the use of coarse meshes, which is important in the analysis of large-scale RC structures. The efficiency of such a modelling approach has been shown earlier by Engen [68] (slightly better agreement with experimental data than for a model prepared according to the Dutch Guidelines [2]) on the level of structural members.

Numerical tests performed within CS2 prove the model’s objectivity, since the dependency on mesh size or load increment is rather irrelevant. The proposed solution strategy reproduced the failure of the beam in bending due to rebar yielding. Despite the simplified description of concrete behaviour in a compressive regime, the proposed model was able to capture beam failures in shear affected by local concrete crushing (CS3 and CS4), as well. Hence, the results presented within CS2–CS4 show the model generality in the NLFEA of full-scale RC members. The summary of performed analyses were shown in Table 2. After validation the proposed model gave very accurate estmation of ultimate load (mean ratio of numerical predictions to experimental results equal 0.998). The bigger discrepancy occurred for the deflection correspond to the ultimate load (mean ratio 1.250). However, for levels of service loads, deflections of the examined RC members were much more precisely predicted.

Significant attention has been paid in the literature to the proper characterisation of concrete behaviour in the post-cracking and post-crushing regime. The author follows the point of view expressed in References [18,22] that, in engineering applications concerning reinforced structures, these issues are not as important as in the case of structures without or with low reinforcement. The most popular method of reducing mesh sensitivity based on the crack band theory proposed by Bazant and Oh [71] (mesh-adjusted softening modulus) for bigger element sizes results in a very steep descending branch (or even snap-back behaviour) [22], which is close to fully brittle behaviour. Conversely, in the case of very fine meshes, models based on these assumptions become unrealistically ductile [49]. Another moot point is the experimental testing of post-cracking behaviour, since it is often difficult to distinguish whether we are measuring material response or the interaction of a sample with test equipment (due to unintended friction) [11,17]. Thus, the brittle modelling of concrete in tension seems to be a reasonable approach. For structures made of plain or lightly reinforced concrete, however, more sophisticated material models may be necessary (with appropriate regularisation technique; see Reference [72]).

RC structures designed according to modern design standards should fail due to rebar yielding, so stress states with high compressive hydrostatic stress are quite rare in real structures. Hence, sophisticated constitutive models, which include phenomena, such as deviatoric-hydrostatic coupling or dilatation, are most often unnecessary. The proposed model seems to be a good compromise. It includes the effect of triaxiality of the stress state in contrast, e.g., to the model proposed in Reference [32] (because modules are related to octahedral shear strain and are prone to concrete crushing, as well). The model needs an appropriate number of load increments, since it is formulated in terms of hypoelasticity with unloading conditions. Conversely, it demands fewer equilibrium iterations, since it produces small values of unbalanced nodal forces in comparison, e.g., to Kotsovos’ material model [17]. Moreover, as mentioned above, the smaller number of increments does not always result in shorter calculation time due to the larger total of iterations necessary [57]. The proposed model is easy to calibrate, since its base is only one material function ($G_{t}$) and does not include parameters with unclear physical interpretation.

The validity of including TS via the constitutive relationship of reinforcing steel was demonstrated in CS2–CS4. The proposed generalised constitutive model of steel is in accordance with the standards [30,31], basic knowledge of RC theory and has clear physical justification. The stiffness of structural members is precisely predicted, especially for the utility load range. It does not introduce another non-linearity and does not demand fine meshes like more sophisticated approaches based on the bond-slip law. The proposed model, however, should be used in the modelling of structures, in which proper anchorage of rebar is provided [73].

The calculations were performed on a personal computer with an Intel i5 M560 processor with basic frequency 2.66 Hz (using 2 cores) and 8 GB RAM memory. The model dimensions, calculation times, iterations and increments needed to find the solution are summarised in Table 3. The obtained calculation times are quite shorter than those reported by Engen [22] and quite longer than those obtained by Markou [18]. The calculation times are not fully comparable due to different computer units used in each research. Engen [22] performed the analysis without parallel processing. Further, the integration of Kotsovos’ material model produces additional unbalanced forces. The proposed material model for compression demands the use of shorter load increments, but the simple integration rule results in the quicker realisation of the equilibrium iteration. Markou [18] developed software dedicated to the analysis of RC structures with a numerically modified Kotsovos’ material model, so that his code was optimised to solve this specific problem. All of the above-mentioned solution strategies, however, could be considered as numerically efficient and applicable to the analysis of large-scale RC structures in comparison with sophisticated elasto-brittle-plastic models (see examples in Reference [18]). The calculation times for very fine meshes, in which the dimension is smaller than recommended in Section 2.5, are much longer than for coarser meshes. Furthermore, one can clearly see that calculation time strongly depends on complexity of analysed problem, not only on the its dimensions (much longer execution times for shear failure modes).

## 5. Conclusions and Future Work

The model complexity and level of details are connected with the area of research and design interest. The aim of the present work is the suitable prediction of the ultimate load capacity and deformability of RC structural members and structures with NLFEA for engineering applications. The models for such analyses should not only ensure good agreement with experimental findings but be easily calibrated and also numerically effective. The presented results show that the proposed solution strategy, in which the primary parts are new constitutive models of concrete and steel, allows to obtain accurate results in relatively short time, thus fulfilling these basic demands. The proposed hypoelastic-brittle material model for concrete is easy to calibrate and allows the use of coarse meshes. Such fully-brittle material model for concrete based on Reference *PJ* criterion was not reported in the literature before. Most of other recent fully-brittle materials model for concrete neglects the TS effect [18,22]. The major novelty of proposed approach is that the TS effect was included by the generalised constitutive model of reinforcing steel in accordance with the fib Model Code 2010 standard [31]. This approach has strong physical background, since it follows from the equilibrium equations of rebar in concrete cover. Consequently, it allows to predict RC member deflections accurately. Moreover, the proposed model was coded as UMAT user’s procedure, which can be also used in open-access software (e.g., CalculiX [74]).

Further studies, associated with the proposed solution strategy, will concern the incorporation of the effects of long load duration times (mostly shrinkage and creep), due to the refined approach to elements deflection. This allows the assessment of serviceability limit states with advanced numerical tools. Another issue which will be examined in the future is the estimation of the reliability characteristics of RC structures. The another possible way of the proposed model development is to adjust it to non-monotonic loads, which requires the improvement of crack-closure algorithm [20] and introduction of damage factors [21]. This would enable an assessment of RC structures resistance under seismic loads.

## Figures and Tables

**Table 1:** Parameters of proposed models adopted in case studies.

No	Parameter	CS1	CS2	CS3	CS4
Concrete model					
1	$E_{c}$ (GPa)	28	29	28	28
2	ν[1]	0.2	0.2	0.2	0.2
3	$r_{\sigma }$ [1]	0.2	0.2	0.2	0.2
4	$\rho_{p}$ [1]	−0.08	−0.05	−0.05	−0.005
5	$f_{c}$ (MPa)	24.0	27.0	22.5	24.1
6	$f_{t}$ (MPa)	2.0	3.2	2.2	2.5
Tension stiffening model					
7	$E_{s}$ (GPa)	-	196	200	200
8	$E_{t}$ (GPa)	-	10	10	10
9	$f_{y}$ (MPa)	-	500	555	555
10	α [1]	-	1.1	1.1	1.1
11	β [1]	-	0.4	0.4	0.4
12	$\rho_{eff}$ [1]	-	0.017	0.035	0.035

**Table 2:** Summary of presented case studies; accuracy of ultimate force prediction (*F*) and deflection corresponding to it (*u*).

CS	$F_{\exp}$ [kN]	$F_{\mathrm{NLFEA}}$ [kN]	$\frac{F_{\mathrm{NLFEA}}}{F_{\exp}}$ [1]	$u_{\exp}$ [mm]	$u_{\mathrm{NLFEA}}$ [mm]	$\frac{u_{\mathrm{NLFEA}}}{u_{\exp}}$ [1]
2	41.0	40.9	0.998	10.0	12.0	1.200
3	330.0	329.0	0.988	6.6	8.9	1.348
4	467.3	471.2	1.008	13.8	16.6	1.203
mean			0.998			1.250
CoV			0.008			0.057

**Table 3:** Models dimensions and calculation times for CSs.

	Mesh	CS2	CS3	CS4
dimensions of model				
variables	coarse	690	1572	1980
	medium	1536	2160	2616
	fine	2451	4443	5085
	very fine	8781	14,730	16,818
equations	coarse	420	1320	1320
	medium	963	1848	1848
	fine	1755	4005	4005
	very fine	7440	13,950	13,950
calculation effort				
increments	coarse	50	58	66
	medium	50	57	55
	fine	70	82	87
	very fine	145	216	262
iterations	coarse	129	676	691
	medium	231	709	450
	fine	443	930	1046
	very fine	1389	2496	3214
calculation time [s]	coarse	14	107	114
	medium	28	204	57
	fine	70	361	276
	very fine	770	1660	2130

## Data Availability

The data presented in this study are available on request from the author.
